# HPV genotypes co-infections associated with cervical carcinoma: Special focus on phylogenetically related and non-vaccine targeted genotypes

**DOI:** 10.1371/journal.pone.0187844

**Published:** 2017-11-21

**Authors:** Rashmirani Senapati, Bhagyalaxmi Nayak, Shantanu Kumar Kar, Bhagirathi Dwibedi

**Affiliations:** 1 Viral Research and Diagnostic Laboratory, Regional Medical research Centre (ICMR), Nalcosquare, Chandrasekharpur, Bhubanewar, Odisha, India; 2 Department of gynecology oncology, Acharya Hari Hara Regional cancer centre, Cuttack, Odisha, India; 3 Directorate of Medical research, IMS & SUM Hospital, S 'O' A University, Bhubaneswar, Odisha, India; Universidade Estadual de Maringa, BRAZIL

## Abstract

HPV is the major causative agent for cervical cancer. Study on the risk of cervical cancer associated with different hr-HPV genotypes would be useful for disease management and new vaccine strategy. With limited reports available, the present study aimed to investigate the pattern of HPV genotypes coinfections and risk of cervical carcinoma associated with them in Indian population. 15 HPV genotypes were detected by E6/E7 multiplex nested type-specific PCR in the HPV-positive cervical samples of 172 cervical cancer cases and 174 subjects with normal cytology. Association between the genotypes and cervical cancer was estimated by calculating the Odds ratio and 95% confidence interval. Risk of cervical carcinoma was associated with multiple genotypes excluding HPV16 (OR:5.87; 95% CI-1.28-26-29; p = .02), multiple genotypes excluding HPV18 (OR = 2.5; 95% CI = 1.09–6.05; p = .03), multiple genotypes of α9 species(OR = 5.3 95% CI = 1.14–24.03; p = .007), and multiple genotypes of α7 species (OR = 2.5; 95% CI = .49–13.45; p = .2). Genotypes not targeted by quadrivalent vaccine types (OR = 2.94 95% CI = 1.48–5.80; p = .001) conferred 2.94 fold higher risk of cervical carcinoma. Cases those coinfected with phylogenetically related genotypes (OR = 2.29; 95% CI(.69–7.59) p = .17) were at 2.9 fold higher risk of invasive cervical carcinoma than those infected with other genotypes although it is not statistically significant. Whereas phylogenetically unrelated genotypes coinfection is negatively associated with cervical carcinoma (OR = .44 95% CI (.244-.8) p = .007) and it is statistically significant.Genotypes not targeted by 9-valent vaccines (OR = .40; 95% CI = .19-.85; p = .017) associated with lesser risk of cervical carcinoma as compared to other genotypes. Subjects infected with any HPV genotype/genotypes excluding HPV16 in association with HPV 18 (OR = 4.1; 95% CI = 1.81–9.25 P = < .001) were at 4.1 fold higher risk of developing invasive cervical carcinoma.In conclusion, the risk of development of cervical cancer is genotype specific and might be associated with type-specific interactions between the genotypes in multiple infections.

## Introduction

HPV infection is the major cause of cervical cancer. HPV 16 and 18 are the most prevalent carcinogenic hr-HPV types associated with 70% of the high-grade cervical lesion and targeted by the current vaccines such as Gardasil and Cervarix [1]. HPV types 31, 33, 45, 52 and 58 are covered in Gardasil 9-Valent vaccine (Human Papillomavirus 9-valent Vaccine, Recombinant) which has recently been approved by the U.S. Food and Drug Administration [2].

The important genital HPV genotypes belong to the genera of alpha(α) papillomaviruses. The members of α -9 species show distinct genomic sequences and similar pathological properties resembling with HPV16 genotype due to their phylogenetically relatedness. α-9 includes HPV types 16,31, 33, 35, 52, 58 and 67. Viruses of α- 7 species resembles with HPV 18 which includes HPV types 39, 45, 59, 68, 70 and 85 along with HPV 18 [3]. Cross protection provided by the bivalent and quadrivalent vaccine to phylogenetically related genotypes have been evaluated by few studies [4,5]. These phylogenetically related clads are expected to have a synergistic action on the progression of the cervical lesion. But there is a little evidence on it.

Cross-protection of the bivalent vaccine (Cervarix) and the quadrivalent vaccine against HPV 31, 33 and 45 types have been reported [5–10]. Theoretically, prevalence of HPV types not targeted by the vaccine could increase or decrease by vaccination which is otherwise called type replacement [11, 12]. But, there is a little evidence of whether type replacement occurs due to vaccination[13–15]. Evaluating the risk associated with non-vaccine targeted genotypes is important for diseases management and devising the second generation vaccine strategy.

Cervical cancer is often associated with multiple HPV genotype infection. Thus it is considered that co-infection may be a risk factor that drives carcinogenesis [15, 16].But contradicting studies invoke confusion about the correlation between multiple infection and cervical disease. It is yet to be understood that whether the risk of cervical cancer is dependent on any typespecific combination or is it common for all the genotypes coinfections? Whether any type specific interaction between the genotypes in multiple infections that increase the risk of cervical cancer development? These questions are more important for the future impact of vaccination and disease management. It is reported that susceptibility to cervical cancer depends on the host genetic factors such as HLA polymorphism which varies with the different ethnical group [17–21]. Hence, the study on the type-specific association of cervical cancer in a different ethnic group is essential. The present study aimed to estimate the association between invasive cervical carcinoma and co-infections with specific HPV species and genotypes in female Indian population.

## Methods

### Study population and sample collection

Suspected cases of cervical cancer attending OPD of Acharya Hari Hara Regional Cancer Center and SCB medical college, Cuttack, Odisha between January 2014 and December 2015 have been enrolled in the study. Married women, above 18 years showing any of the symptoms like abnormal vaginal bleeding/discharge, pain during coitus, lower abdominal pain and clinician suspicion of cervical malignancy were included in the study after clinical examination by a gynecologist. Unmarried women, pregnant cases and patients undergoing treatment were excluded. Cases and control were identified on the basis of cytology.

Informed written consent to participate in the study was obtained from all the enrolled cases. This study is approved by institutional ethics committee of Regional Medical Research Center (ICMR), Bhubaneswar, Odisha, India.

Clinical data including signs and symptoms and socio-demographic data such as age, education, economic status, age at marriage, the age of menopause etc were collected by interviewing the patients with a predesigned questionnaire.

Cervical swab specimen was collected using cytobrush and stored inside the viral transport media (Hi-Media) and transported to Virology laboratory, Regional medical research center, Bhubaneswar, Odisha for further analysis. Pap smears were prepared from the collected cervical sample for cytological analysis. Cytological classification was done according to Bethesda system [10].

### HPV genotyping

DNA was extracted from a 200μl aliquot of exfoliated suspended cell samples using the QIA amp DNA Blood Mini Kit (Qiagen) as per the manufacturer’s instructions. Amplification of the human β-globin gene was performed to test sample sufficiency. To detect HPV, PCR was performed by using PGMY09/PGMY11 primers (MY11: 50-GCMCAGGGWCATAAYAATGG-30; MY09: 50-CGTCCMARRGGAWACTGATC-30–) targeting a 450-bp region of the HPV L1 gene. E6/E7 Type specific primers were used for the detection of 15 HPV genotypes. Type-specific primers were chosen according to the previously published study [22].

The PCR reaction mixture containing 10X Taq buffer (Biotools, Spain), 250mM dNTP (Biotools, Spain), 5U Taq DNA polymerase (Biotools, Spain), primers and 50ng of DNA was processed for the first PCR cycle. The temperature conditions were: 4 min of an initial denaturation step at 94°C, followed by 35 amplification cycle. Each cycle includes the 30s of denaturation at 94°C, 30s of annealing at 56°C, elongation at 72°C for 45s and a final extension of 4 min at 72°C. First PCR was followed by nested type-specific PCR. 2μl of the PCR product was used for the nested PCRs. PCR product was analyzed by electrophoresis on 2% agarose gel stained with ethidium bromide followed by visualizing under UV light.

### Statistical analysis

This is a case control study. Infection with more than 1genotypes was considered as multiple infections. In triple/quadruple genotype combinations only those having all the three genotypes of different species were considered as phylogenetically unrelated clad. Odds ratio were calculated for estimating the association of genotypes with cervical carcinoma. Statistical analyses were performed by using MediCal version 14.10.2 [23]. A p-value of ≤0.05 was considered as statistically significant.

## Results

**Fig 1:**
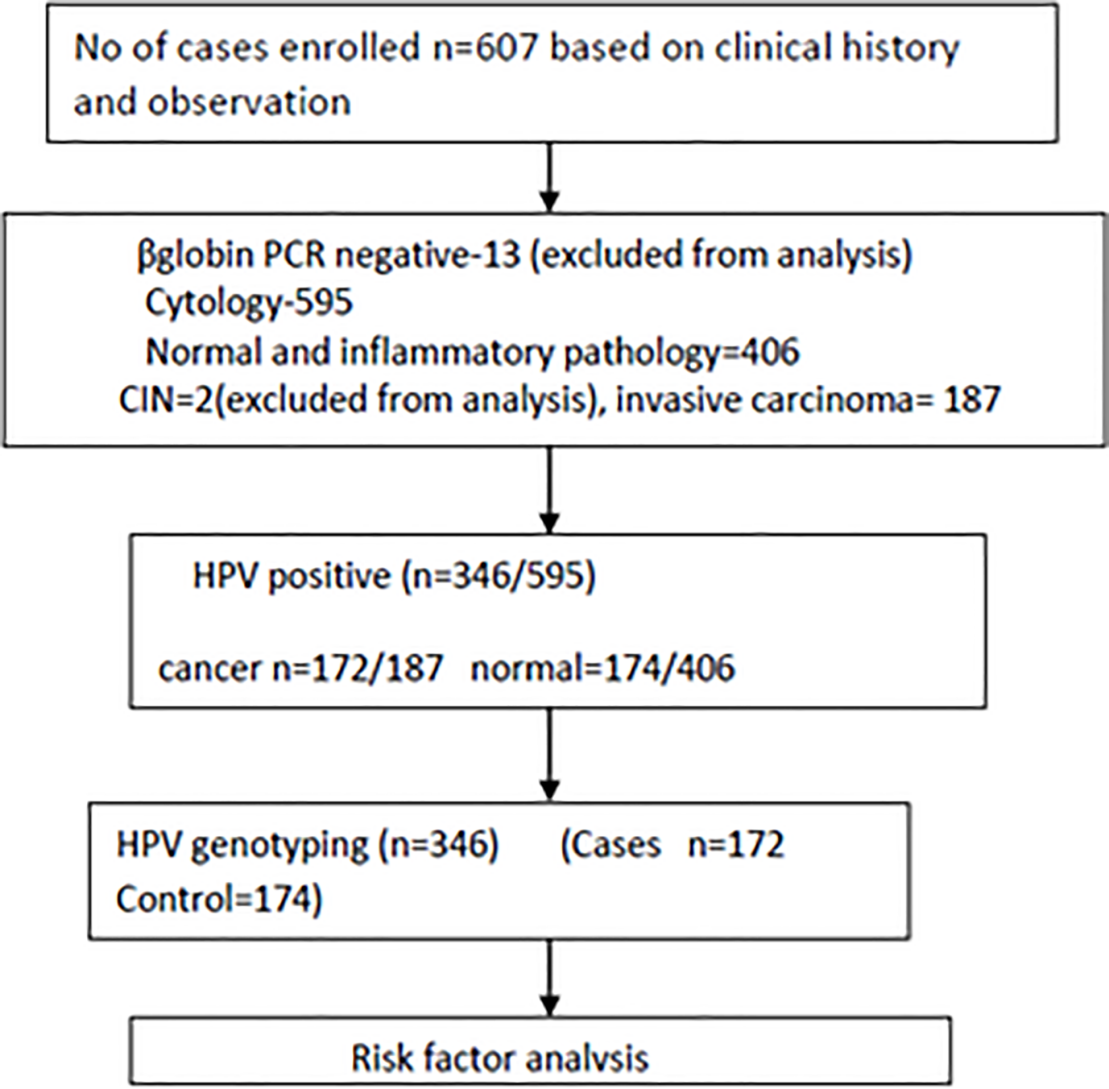
Enrollment of cases and outcomes.

Subject enrollment and outcomes are presented in Fig 1. A total of 607 subjects have been enrolled in this study. Out of which 13 subjects excluded as they were negative for βglobin PCR which determines sample sufficiency. 595 samples were analyzed for cytology and HPV PCR. Only 346 cases were found to be positive for HPV. Out of which172 wereconfirmed cases of cervical cancer and 174 with normal cytology having minor gynecological symptoms such as abnormal discharge, bleeding and pain during coitus, lower abdominal pain, intermenstrual bleedingconsidered as controls in the study.

346 HPV positive samples were genotyped for 15 genotypes of HPV. Out of 346 samples 172 cases (Mean-44.7; Median-45; Range- 19–86 years) were invasive cancer and 174 cases (Mean-55.16; Median-55; Range 25–86 years) without abnormal cytology. The genotypes detected were HPV16,18,51,39,66,68,45,35,6/11,58,43 and 42.Prevalence of multiple genotypes was 23.41% with double, triple and quadruple genotypes while the prevalence of single genotype infection was 76.59%.The study population was positive for 6 species (α5, 6, 7, 8, 9 and 10) of HPV genotypes (Table 1). Among both normal and the cervical carcinoma cases more than 90% cases were infected with α -9 HPV. Prevalence of α 6 and 7 was higher among the cases with normal cytology as compared to invasive cervical carcinoma cases whereas it is reverse incase of α-5.No normal cases were found to be infected with α -6, 8 and10.

**Table 1:** Distribution of cases infected with different species of HPV genotypes.

HPV status	Cervical carcinoma cases(Cases)	Normal cases (Control)	P value
HPV Positive	172/172	174/406	.0001
Multiple genotypes	39/172	49/174	.204
Alpha 5 (HPV 51)	5/172(2.9)	3/174(1.7)	.70
Alpha 6(HPV66)	4/172(2.3)	6/174(3.4)	.78
Alpha7(HPV18,39,68,45)	42/172(24.4)	55/174(31.6)	.17
Alpha 8(HPV 43)	2/172	0	.47
Alpha 9 (HPV16,45,58,35,52)	157/172(91.27)	157/174(90.22)	.87
Alpha 10(HPV 44,6/11)	6 /172(3.4)	0	.03

**Fig 2:**
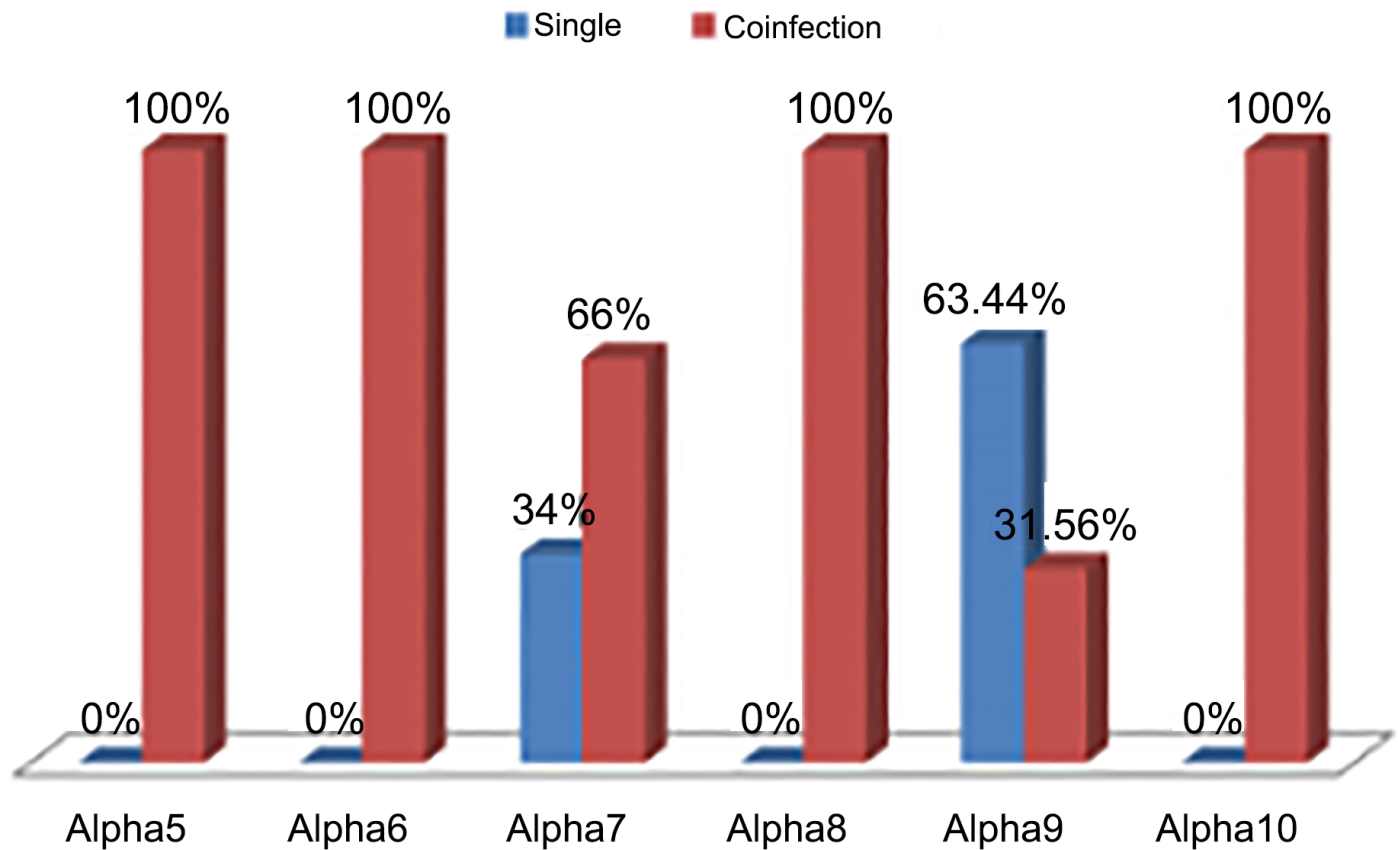
Distribution of HPV genotypes as single and coinfection in different phylogenetic groups.

HPV genotypes belong to α5, α6, α8 and α10 species were always present as co-infection. Genotypes of α7 and α9 species were present both as single and co-infection where co-infection percentages were 66% and 31.56% respectively (Fig 2).

Cases and controls were compared by demographic and other factors(Table 2).It was found that higher age at diagnosis, Parity, age of marriage and Low education were significantly associated with cervical carcinoma.

**Table 2:** Demographic factors associated with cervical carcinoma.

Factors	Cases N = 172(%)	Control N = 174(%)	*P*
Age > 45	152(88.37)	77(44.52)	< .0001
Parity ≥ 3	135(78.48)	74(42.52)	< .0001
Age of marriage > 18	89(51.74)	138(79.31)	< .0001
Low Education	167(98.25)	138(79.31)	< .0001
Low socioeconomic	135(78.48)	123(70.68)	.12
Rural	118(68.6)	120(68.96)	.94
Poor menstrual hygiene	140(81.39)	143(82.18)	.95

The risk of cervical cancer associated with single and multiple infections have been evaluated (Table 3). There was no association of cervical carcinoma with overall multiple or single infection. However, multiple infections with specific genotypes were associated with cervical carcinoma. Overall multiple infections excluding HPV16 (OR = 5.87; 95% CI-1.28-26-29;p = .02) and HPV 18(OR = 2.5; 95% CI = 1.09–6.05;p = .03) were significantly associated with cervical carcinoma. Women with multiple genotypes of α9 species had a 5.3 fold risk of cervical cancer (OR = 5.3 95% CI = 1.14–24.03; p = .007). Multiple genotypes of α7 species had 2.5 fold risk to cervical cancer (OR = 2.5; 95% CI = .49–13.45; p = .2).

**Table 3:** Association of multiple infections with the risk of cervical cancer.

Single /Multiple	Cancer(Cases) n = 172(%)	Normal(Control) n = 174(%)	OR(95% CI)	P
Multiple n = 81	39(22.67)	42(24.13)	.92(.56–1.51)	.74
Single n = 265	133(77)	132(75.86)	1.08(.95–1.78)	.74
**HPV 16**				
Multiple	57(33.13)	57(32.75)	1.01(.64–1.59)	.93
Single	115(66)	117(67.24)	.98(.62–1.59)	.93
**HPV 18**				
Multiple	20(11.62)	34(19.54)	.54(.29-.98)	.04
Single	16(9.30)	15(8.62)	1.08(.51–2.27)	.82
**Excluding HPV 16**				
Multiple	11(6.39)	2(1.14)	**5.87(1.28-26-29)**	**.02**
Single	18(10.46)	15(8.62)	1.2(.60–2.54)	.55
**Excluding HPV18**				
Multiple	19(11.04)	8(4.59)	**2.57(1.09–6.05)**	**.03**
Single	117(68.02)	117(67.24)	1.03(.66–1.62)	.87
**Alpha 9**				
Multiple	10(5.81)	2(1.14)	**5.3(1.14–24.59)**	**.03**
Single	115(66.86)	117(67.24)	.98(.62–1.59)	.93
**Alpha 7**				
Multiple n = 7	5(2.90)	2(1.14)	**2.5(.49–13.45)**	0.2
Single(HPV18,45)	18(10.46	15(8.62)	1.23 (.60–2.54)	.55
Any Phylogenetically related coinfection	9(5.23)	4(2.29)	2.29(.69–7.59)	.17
Any Phylogenetically unrelated coinfection	19(11.04)	38(21.83)	**.44(.244-.80)**	**.007**
Non Vaccine (4v) targeted in associated/presence of any vaccine targeted (45/35/51/39/44/43/68/66 without or with 16/18/6/11)	33(19.18)	13(7.47)	**2.94(1.48–5.80)**	**.001**
Non Vaccine (9v) targeted in associated/presence of any vaccine targeted (35/51/39/44/43/68/66 without or with 16/18/11/6/31/33/45/58/52)	11(6.39)	25(14.36)	**.40(.193-.856)**	**.017**
HPV16+18	8(4.65)	29(16.66)	**.25(.11-.56)**	**.000**
Any single/multiple(16/18/other type single or multiple/16+18+other genotype) type except HPV16+18 combination	164(95.34)	145(83.33)	**4.1(1.81–9.25)**	**.0007**
Alpha 9	125(72.67)	119(68.39)	1.22(.77–1.95)	.38
Alpha 7	23(13.37)	17(9.77)	1.4 (.73–2.77)	.29

(Normal: These are the cases with normal cytology(including inflammatory cytology) having minor gynecological symptoms such as abnormal discharge, bleeding and pain during coitus, lower abdominal pain, intermenstrual bleeding considered as controls in the study).

Genotypes not targeted by quadrivalent vaccine types (OR = 2.94 95% CI = 1.48–5.80; p = .001) conferred 2.94 fold higher risk of cervical carcinoma. Cases those coinfected with phylogenetically related genotypes (OR = 2.29; 95% CI(.69–7.59 p = .17) were at 2.9 fold higher risk of invasive cervical carcinoma than those infected with other genotypes although it is not statistically significant. Whereas phylogenetically unrelated genotypes coinfection is negatively associated with cervical carcinoma (OR = .44 95% CI (.244-.8) p = .007)and it is statistically significant. Genotypes not targeted by 9-valent vaccines (OR = .40; 95% CI = .19-.85; p = .017) associated with lesser risk of cervical carcinoma as compared to other genotypes. Subjects infected with any HPV genotype/genotypes excluding HPV16 in association with HPV 18 (OR = 4.1; 95% CI = 1.81–9.25 P = < .001) were at 4.1 fold higher risk of developing invasive cervical carcinoma.

## Discussion

To the best of our knowledge, this is the first study to address the association of risk of cervical cancer with specific HPV genotypes coinfection among the Indian population.However the study population was drawn from tertiary care hospital and not from community screening. The present study has demonstrated many meaningful facts that could have profound implications for disease management and devising new vaccine strategy.

The present study reported thathigher age at diagnosis, multiple parity, early age of marriage and low education found to be significantly associated with cervical carcinoma. This is in consistence with other previously reported studies [24,25].

The relationship between multiple HPV type infections and the progression of cervical cancer is debatable. However, competitive or cooperative interactions between HPV types could increase the risk of progression to cervical cancer. The present study reveals that women infected with multiple genotypes with phylogenetically related clad had the higher risk of cervical carcinoma as compared to the population infected with phylogenetically unrelated clad. Multiple infections with alpha-9 genotypes conferred asignificantly 5.3 fold higher risk while coinfection with alpha-7 genotypes conferred 2.5 fold risk of cervical cancer. It might explain a synergistic action of the same species of HPV genotypes when coinfected together for the induction of cervical cancer. Prevalence of Alpha 9 species is shown to be higher than alpha-7 is in agreement with other studies [26]. Our study also shows a comparatively higher carcinogenic potential of alpha-9 species than the alpha-7. In a similar study Chaturvedi et al demonstrated that women with multiple infections were at significantly increased risk of both CIN2+ and HSIL+ when compared with single infection, with the highest risk being in those having multiple oncogenic types of α-9 species [15].

Interactions between different HPV types during their life cycle may affect the propagation, pathogenesis, and evolution of HPVs [27].The present study shows that HPV 16 and 18 when co-infected together in absence of other HPV types, may have the low risk of cervical cancer as compared with other genotypes as single or multiple. It might explain their phylogenetically unrelatedness and thus the antagonistic action of HPV16 and 18 when present together in a coinfection. *In vitro* study reveals that co-infection of a single cell with both HPV16 and HPV18 results in replication interference between them [27]. Several pathological studies also demonstrated the interference between multiple HPV types. Seropositivities for HPV6 and HPV11 significantly antagonize the development of HPV16-related cervical cancer [28, 29]. Antagonistic interactions between HPV16 and HPV18 have also been reported by the same study. However, the influence of other genotypes on the interference of genome replication between HPV16 and HPV 18 types has not yet been understood.

HPV vaccination may not have a profound impact on non-vaccine targeted genotypes [15]. Hence the risk of cervical diseases with non-vaccine targeted types was assessed in this study. Women infected with genotypes not targeted by quadrivalent vaccines were at 2.9 fold higher risk of developing invasive cervical carcinoma whereas genotypes not targeted by nonavalent vaccine were at lesser risk. It explains the maximum effectiveness of 9v-vaccine covering 9 important high-risk genotypes.HPV51 and HPV58 may develop carcinogenesis more rapidly and more extensively when infected with other HPV types [30]. Chagas et al 2015 reported the association of NV type HPV 56 with the risk of cancer development [31]. Munagala 2009 reported that the NV genotypes associated with poor prognosis [32].

The present study reported that most of the non-vaccine targeted genotypes were co-infected with HPV16/ 18 except HPV45 and HPV58 [30]. It was found that HPV45 present as single infection as well as in association with HPV18 in cervical cancer cases. The association of HPV45 with invasive cancer without the coinfecting with HPV16 and 18 throws light on its high oncogenic potential [1]. Our findings support other studies [33] in considering HPV 58 to be a highly oncogenic genotype for its association with cervical cancer in the absence of HPV16/18.

Present data shows that genotypes from certain species (α5, 8 and 10) appear as co-infecting more frequently than genotypes from other species (α9 and 7). These findings may be indicative of differential dependency for each genotype or species to colonize cervical epithelium. However, the strength of the association and whether this probable dependency is intra or interspecies need to be addressed with large sample size. These results may support the theory of elimination of certain genotypes by vaccination may affect the distribution of other genotypes or the theory of type replacement. In conclusion, the risk of development of cervical cancer is not common for all the genotypes. There might be type specific interactions (synergistic/antagonistic) between the genotypes in multiple infections that increases or decreases the risk of development of cervical cancer. The preliminary findings of the present study suggest further longitudinal study with large population size to shed more light on the carcinogenic potential of the HPV genotypes.
